# Effects of a Cannabinoid-Based Phytocomplex (Pain Relief^TM^) on Chronic Pain in Osteoarthritic Dogs

**DOI:** 10.3390/ani15010101

**Published:** 2025-01-05

**Authors:** Ruggero Amato, Eleonora Pacifico, Daria Lotito, Valeria Iervolino, Ludovica Pierantoni, Laura Cortese, Nadia Musco, Pietro Lombardi, Vincenzo Mastellone, Maria Elena Pero

**Affiliations:** 1 Department of Veterinary Medicine and Animal Production, University of Naples Federico II, 80137 Naples, Italy; ruggero.amato@unina.it (R.A.); daria.lotito@unina.it (D.L.); laura.cortese@unina.it (L.C.); nadia.musco@unina.it (N.M.); pilombar@unina.it (P.L.); vincenzo.mastellone@unina.it (V.M.); mp3054@cumc.columbia.edu (M.E.P.); 2 Dipartimento di Medicina Veterinaria e Scienze Animali, University of Milano, 20122 Milan, Italy; eleonora.pacifico@unimi.it; 3 CAN, Comportamento Animale Napoli, 80128 Naples, Italy; ludovica.pierantoni@gmail.com; 4 Department of Pathology, Anatomy and Cell Biology, Columbia University, New York, NY 10032, USA

**Keywords:** dogs, chronic pain, cannabidiol, nutritional supplement

## Abstract

Twenty-one adult crossbreed dogs with chronic pain due to severe osteoarthrosis were enrolled in the study (placebo vs. treatment groups). The dogs in the experimental group received the dietary supplement (Pain Relief^TM^, Giantec, Isernia, Italy) for 30 days to evaluate its effects on metabolism and pain relief. During the trial, the Helsinki Chronic Pain Index significantly decreased (*p* < 0.01) in the experimental group, indicating reduced pain and improved quality of life. Additionally, the treated group showed improvements in oxidative stress, demonstrated by a reduction in reactive oxygen metabolites, and an increase in biological antioxidant potential. Interleukins 6 levels decreased in the treated group, while interleukins 10 levels increased, thus suggesting an anti-inflammatory effect of the supplement. Importantly, no adverse effects were observed. Results suggest that Pain Relief^TM^ is effective in ameliorating osteoarthritis in dogs, improving their quality of life.

## Simple Summary

Chronic pain is one of the most disabling conditions in dogs, as it affects various aspects of a dog’s life and should be managed regardless of the severity of symptoms. This research investigates the effects of a cannabidiol-based nutritional supplement in dogs affected by severe osteoarthritis. The treated group showed a reduction in pain due to an improvement of some cytokines expression and oxidative status. This suggests that Pain Relief^TM^ possesses an anti-inflammatory effect and reduces pain perception in dogs, thereby enhancing their quality of life.

## 1. Introduction

“Pain is a more terrible lord of mankind than even death”—Albert Schweitzer, 1922. The importance of pain in dogs, as well as in humans, can be easily understood considering that the first uses of anesthetic protocols in animals were designed to relieve pain and not to anesthetize the animal. Thus, it can be assumed that pain therapy allowed the birth of anesthesiology [1]. Furthermore, it is important to note that, unlike other drugs that require careful diagnosis, before administration, some authors argue that pain treatment should proceed regardless of the severity of clinical signs [2]. In contrast, there is a growing awareness among pet owners about the risks associated with the excessive use of synthetic drugs, which has fueled interest in natural products, with few or no contraindications that can be used routinely and/or continuously over time. Over the last twenty years, pain research has focused on molecules or compounds that can alleviate both chronic and acute pain in dogs. Additionally, various efforts have been made to assess the biochemical markers related to pain. Among these, analyzing oxidative status, including antioxidant potential, has emerged as one of the most promising methods for evaluating inflammatory states and associated pain, as recommended by the AAHA guidelines [3]. Based on these premises, research has focused on assessing pain and the effectiveness of natural products, showing that food supplements made from natural compounds can effectively reduce oxidative metabolite levels [4]. This not only alleviates pain perception but also minimizes the damage caused by the inflammation itself. Among natural products, cannabis is undoubtedly one of the most popular and widely discussed plants in recent years. Cannabis has undergone a unique process of genetic selection to maximize the concentration of pharmacologically active molecules in its phytocomplex. Currently, the cannabis plant (in its various genotypic and phenotypic variations) and its derivatives are considered as valuable or promising resources for a wide range of conditions, including Alzheimer’s disease, psoriasis [5,6], hair loss [7], cancer, and stress [8,9], and to alleviate peripheral neuropathy symptoms associated with chemotherapy, an adverse sequela of cancer therapy characterized by loss of peripheral nerve function which typically manifests with a distal-to-proximal pattern of progressive peripheral sensory nerve loss. [10,11]. The cannabis-based nutritional supplement tested in this study contains both cannabidiol (CBD) and cannabigerol (CBG), two of the most prevalent compounds in cannabis aside from tetraydrocannabinol (THC). CBD is the primary non-psychoactive cannabinoid with demonstrated pharmacological efficacy [12]. It was the first cannabinoid to be discovered [13] and, alongside THC, has been the focus of much research. While its exact effect on pain and inflammation is not yet fully understood, it appears to involve interaction between CBD and the CB1 and CB2 receptors. CBG is a relatively recently discovered cannabinoid (identified by Yechiel Gaoni in 1964), and in its acid form (CBGA) is the precursor to both CBD and THC. It seems to be particularly involved in pain perception, interacting with both the CB1 and CB2 receptors as well as with cation channels encoded by the TRMP8 gene which transmit the sensation of cold. The supplement also contains Myrcene, a monoterpene (an aromatic molecule in cannabis) that antagonizes TRPV ion channels activated by capsaicin and high temperatures, which are involved in acute pain perception [14]. Additionally, it includes devil’s claw (*Harpagophytum procumbens* root extract), a perennial plant that thrives in arid conditions in Southern Africa. Historically used to address osteo-articular and, in some cases, muscular issues, devil’s claw root, demonstrates anti-inflammatory and pain-relieving properties by increasing the production of catalase and superoxide dismutase [15]. The manufacturer suggests, and the hypothesis is, that the combination of these compounds may reduce chronic pain in dogs, thereby enhancing their quality of life, thanks to the synergic effect of the different molecules combined. To this end, a comprehensive hematological and biochemical screening, along with an assessment of oxidative and inflammatory states was conducted in dogs affected by chronic pain due to severe osteoarthritis and treated with Pain Relief™. It is important to emphasize that the purpose of this research was not to evaluate the efficacy of individual phytocomplexes or molecules within the supplement being studied, but rather to assess the overall effect of Pain Relief™ as a whole.

## 2. Materials and Methods

### 2.1. Nutritional Supplement

The commercial supplement Pain Relief™ (Giantec, Isernia, Italy) is an oil-based formulation (medium-chain triglycerides coconut oil—known for easier absorption in the mucosa and intestine) that combines cannabidiol (15%) and cannabigerol (15%) (with THC levels under 0.2%), myrcene (0.3%), and devil’s claw (1%) alcoholic extract (*Harpagophytum procumbens* subsp. *procumbens* (Burch.) DC. ex Meisn. from the sesame seed family—Pedaliaceae) [16], all diluted in fractionated coconut oil. It is designed to help dogs with both acute and chronic pain.

### 2.2. Animals and Study Design

Twenty-one cross-breed dogs affected by osteoarthritis-related chronic pain (11 males and 10 females; mean age 9.22 ± 1.48 years old) were enrolled with the owners’ consent. The study was conducted on family dogs to avoid any potential interference from environmental changes. All the dogs were fed on a commercial diet for 2 months (including 1 month of adaptation) containing crude protein 211 g kg^−1^, acid hydrolyzed ether extract 90 g kg^−1^ and crude fiber 39 g kg^−1^, 3110 kcal kg^−1^ on a feeding basis. The diet was administered at a ratio of 130 kcal ME per kg 0.75 of ideal body weight. All dogs enrolled had a clinical diagnosis of osteoarthritis-related chronic pain, and only those graded as 3 (severe), according to Pollmeier et al., 2006 [17] were included in the trial.

The Helsinki Chronic Pain Index score [18] was also used. No dogs with comorbidities were included in the trial. After enrollment, the dogs were assigned to two groups (placebo group and treated group) according to a randomized complete block design. The animals did not receive any pharmacological treatment for at least 2 months prior to, and throughout, the trial. Group assignments were not disclosed to the owners. A double-blind design was employed for the study. Pain Relief™ (Giantec srl, Isernia, Italy) was orally administered to the dogs in the treated group at the dosage of 2.5 drops per 10 kg of the body weight (17.5 mg of CBD and 17.5 mg of CBG per 10 kg of live weight) as suggested by the manufacturer. The product was administered for 30 consecutive days, twice daily at approximately 7:00 a.m. and 7:00 p.m. (the half-life of cannabinoids is unclear and often irregular but generally indicated as between 10 and 12 h [19]). The placebo, at the same dosage and in the same packaging, was administered to the dogs in the control group to minimize any false results. In both the treated and placebo groups, the oil was administered directly into the dog’s mouth, after the meal.

### 2.3. Patient Evaluation

A clinical examination and blood analyses were used to determine the health status of all dogs. The same veterinarian performed these procedures, before starting the treatment (day 0) and after 30 days, without knowing which groups the dogs were assigned to. Physical and neurological examinations included assessments of pain on manipulation and palpation, joint swelling, and range of motion, as proposed by Pollmeier et al., 2006 [17]. During the initial evaluation, the most severely affected leg was identified, and ratings for lameness, discomfort on manipulation and palpation, range of motion, and joint swelling were assigned to that leg.

The level of pain during limb manipulation was gauged by observing the animals’ vocalizations, other psychomotor changes, or expressions of pain during brief extension and flexion of all four limbs [20]. Only dogs with extreme pain scores (severe) were included in this trial.

### 2.4. Blood Sampling and Analyses

Blood samples were collected from the fasting dogs in both groups, before and after 30 days of the supplement administration, from the jugular vein into tubes with and without K3-EDTA, and were immediately transported to the laboratory. Serum was obtained by centrifugation at 1200× *g* for 15 min, divided into aliquots, and frozen at −80 °C.

A complete cell blood count was performed for each whole blood sample within 30 min of the collection by a Lasercyte™ haematology analyser (IDEXX Laboratories Inc., Westbrook, ME, USA) that provides complete blood counts: white blood cells (WBC), red blood cells (RBC), hemoglobin (HGB), hematocrit (HCT), mean corpuscular volume (MCV), mean corpuscular hemoglobin concentration (MCHC), platelet (PLT), mean platelet volume (MPV), blood cell distribution width (RDW), lymphocytes (LYM), monocytes (MON), and granulocytes (GRA).

Blood chemistry analyses on serum aliquots were performed by an automatic biochemical analyser (AMS Autolab, Diamond Diagnostics, USA) using reagents from Spinreact (Girona, Spain) to determine: total proteins (TP), albumin (ALB), creatinine (CREA), glucose (GLU), aspartate amino transferase (AST), alanine-aminotransferase (ALT), gamma-glutamyl transferase (GGT), bilirubin (BIL), alkaline phosphatase (ALP), cholesterol (CHOL), triglycerides (TRI), chlorine (Cl), sodium (Na), potassium (K), ionized calcium (Ca^2+^), calcium (Ca), and phosphorus (P).

To assess the potential impact of Pain Relief on oxidative status, the reactive oxygen metabolites derivates (d-ROMs) and biological antioxidant potential (BAP) were measured in serum samples. d-ROMS evaluate free alcohoxyl and hydroperoxyl radicals derived from hydroperoxides in the sample, while BAP measures the chemically active antioxidant capacity of the plasma barrier. Reagents from Diacron International s.r.l. (Grosseto, Italy), validated for use in canine species, were used for the assessments [21]. The inflammatory status was evaluated by measuring Canine Interleukins 6 (IL-6) and 10 (IL-10) and Canine Tumor Necrosis Factor alpha (TNF-α) in serum, utilizing Elisa kits from Genorise (Philadelphia, PA, USA). The TNF-α detection range assay was 1–2200 pg/mL with intra- and interassay CV < 7 and <9%, respectively. The IL-6 detection range assay was 50–3200 pg/mL and 25–1600 pg/mL for IL-6 and IL-10, respectively, with intra- and interassay CV < 6 and <9%, respectively.

### 2.5. Statistical Analysis

Data were statistically analyzed using the non-parametric Mann–Whitney U Test. All the statistical procedures were performed used JMP software version n. 9 (SAS Institute, Cary, NC, USA).

## 3. Results

Only significant results were reported.

### Clinical Evaluation

**Figure 1:**
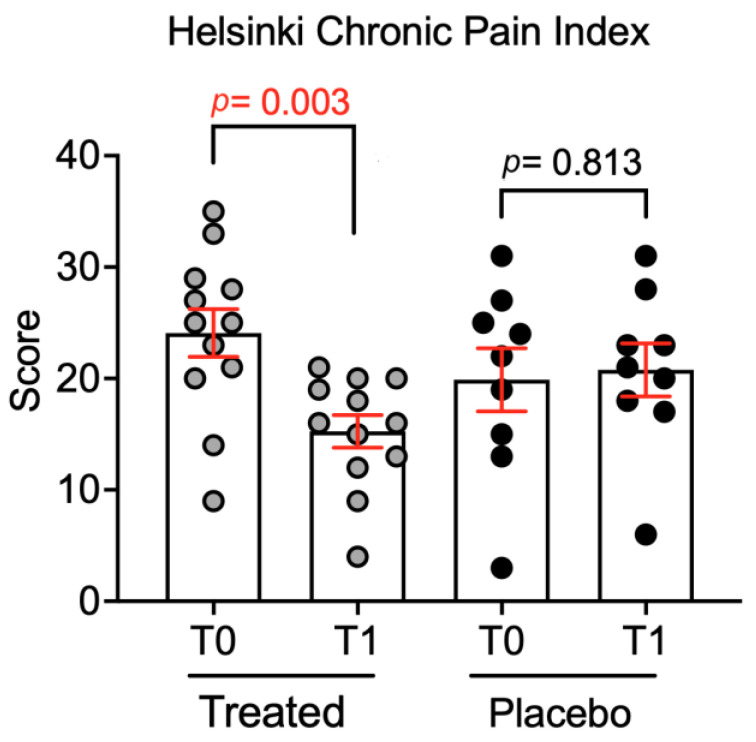
The Helsinki Chronic Pain Index score following Pain Relief administration in the treated (n = 12) and placebo (n = 9) groups is presented. All data are expressed as standard errors, and statistical analysis was performed by using Mann–Whitney U Test (*p* = 0.003).

The treated group showed a significantly lower Helsinki Chronic Pain Index (*p* < 0.01) (Figure 1). Despite the veterinarian having observed an apparent improvement of lameness among treated dogs, they were still classified as severely affected by osteoarthritis according to the Pollmeier evaluation, suggesting that the long-term efficacy of the treatment for osteoarthritis has yet to be demonstrated.

The supplementation was well-tolerated, as all hematological and biochemistry parameters remained within the normal range for adult dogs, and no negative effects were observed during the clinical examination.

**Figure 2:**
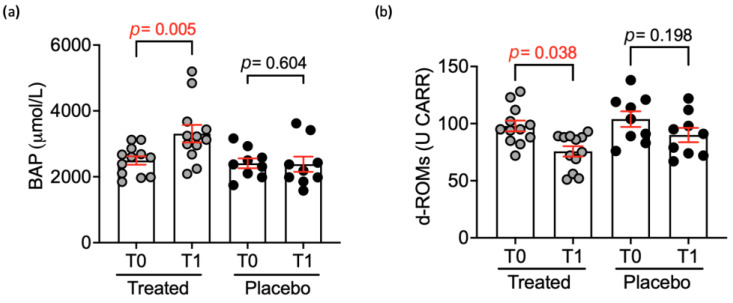
BAP (**a**) and d-ROMs (**b**) levels after 30 days’ administration of Pain Relief in the treated (n = 12) and placebo (n = 9) groups. All data are expressed as mean ± standard error, and statistical analysis was performed by using Mann–Whitney U Test (BAP in treated *p* = 0.005; d-ROMS in treated *p* = 0.038).

Regarding the oxidative status (Figure 2) the d-ROMs significantly decreased (*p* < 0.01), compared to the placebo group, in the treatment group for both the time and the group impact. Likewise, a significant (*p* < 0.05) increase in the BAP levels indicated an improvement in the biological antioxidant potential.

The IL-6 significantly (*p* < 0.01) decreased in the treated group after 30 days of supplementation. On the contrary, the IL-10 significantly (*p* < 0.05) increased. No differences were observed for the TNF-α. Concerning blood chemistry, the CHOL resulted as being significantly (*p* < 0.01) lower in the treated group, whereas no differences were seen for all the other parameters.

## 4. Discussion

Chronic pain is one of the most disabling conditions in dogs as it affects various aspects of a dog’s life, including: reduced sociability and play, altered posture, changes in gait, such as stiffness, lameness or stumbling, as well as hesitation, reluctance or refusal to engage in activities such as jumping into a car, and an overall decrease in activity levels, compared to the dog’s normal behavior [22]. Chronic pain is such a common condition that it has been suggested (in humans) that pain should be a monitored as a vital sign, like pulse and respiration [23]. Our first hypothesis, that the combination of Pain Relief compounds could reduce chronic pain in dogs and improve their quality of life, was confirmed.

### 4.1. Clinical Examination

Based also on the neurological examination, we observed that, at the reported dosage, the Pain Relief™ caused no adverse effects. These results were drawn from two clinical evaluations at T0 and T30 that clearly indicate Pain Relief produced no physical side effects and no signs of intolerance or digestive issues. This partially contrasts with the results by McGrath et al. (2018) [24], who reported CBD to be generally well-tolerated, but demonstrating an increase in cases of diarrhea and a significant increase in ALP. Moreover, in addition to the significantly lower Helsinki Pain Index (*p* < 0.01) in treated dogs, despite all dogs still resulting as severe for the Pollmeier score, we observed an improvement in lameness, discomfort during manipulation and palpation, range of motion, and joint swelling, confirming the findings of Mosley et al. [25].

### 4.2. Pain Perception

**Figure 3:**
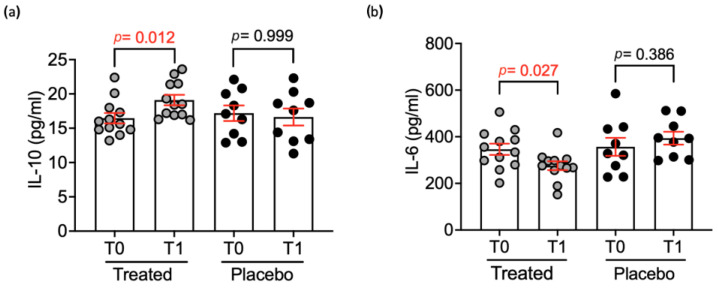
IL-10 (**a**) and IL-6 (**b**) levels after 30 days administration of Pain Relief in the treated (n = 12) and placebo (n = 9) groups. All data are expressed as mean ± standard error, and statistical analysis was performed by using Mann–Whitney U Test (IL-10 in treated *p* = 0.012; IL-6 in treated *p* = 0.027).

The first hypothesis of this study was to determine whether Pain Relief could improve the clinical and biochemical indicators of pain by primarily targeting inflammatory mediators and the oxidative state as shown in Figure 2 and Figure 3. Nevertheless, the previously mentioned effect on pain perception is also associated with the direct impact of cannabinoids on CB1 nociceptors. In fact, it is established that cannabinoids can directly influence the initial stage of pain perception by inhibiting pain signals at the level of the peripheral nervous system [26].

However, it is interesting to note that the effect of cannabinoids may also be linked to an ‘indirect’ action on nociceptors. Specifically, cannabinoids reduce prostaglandin production, which in turn leads to diminished activation of ‘silent nociceptors’ -nociceptors that are not activated until an inflammatory stimulus occurs [27]. The overall result is a decreased perception of pain [28]. Despite being present in low concentration within the phytocomplex, the effect may also be attributed to the iridoid glycosides of devil’s claw, a large group of phytochemical compounds belonging to the monoterpene family (such as myrcene). While the exact mechanism by which these glycosides influence pain perception remains unclear (their interaction with nociceptors is not fully understood) [29], they are known to exert an ‘indirect action’, by reducing oxidation and inflammation, thereby influencing pain perception. Based on this, it can be logically inferred that the lower Helsinki Chronic Pain Index observed in the treated group may be linked to the effect of these compounds on nociceptors and related receptors.

### 4.3. Oxidative Asset and Inflammation Response

The results of blood tests showed a decrease in d-ROMs (*p* < 0.01) and an increase in BAP levels (*p* < 0.05) in the treated group. This reduction could be related to the altered activity of two key enzymes involved in the formation of superoxide radicals, xanthine oxidase (XO) and NADPH oxidase (NOX1 and NOX4) [30]. However, it is not entirely clear if this is the only way cannabinoids exert their antioxidant potential. Another hypothesis is that cannabinoids may modify mitochondrial metabolism, thereby reducing the production of mitochondrial reactive oxygen species [31].

Ultimately, the product acts as hypothesized, reducing both pain and oxidation levels and, as further confirmed by the decrease in Il-6 (*p* < 0.01) and increase in Il-10 (*p* < 0.05), demonstrates a suggestive anti-inflammatory effect. In particular, the effect on IL-6 was previously reported for CBD by Sermet et al. (2021) [32] and in the same year for CBG by Stone et al. (2021) [33]. The reason for this reduction is probably due to the immunomodulation of monocytes by CBD, which results in a lower release of IL-6 into the blood [32]. It is unclear how CBG acts on IL-6, but a CBD-like action on IL-6 produced by astrocytes has been reported [33]. These results align with the literature: Anand et al. (2021) [34] and Arthur et al. (2024) [35] report that both CBD and CBG help reduce inflammation and pain, while Pagano et al. (2023) [36] confirm that cannabinoids may improve the antioxidant state of users. In addition, devil’s claw root, in various forms (most commonly as powder or alcoholic extract) has long been known for its empirically recognized anti-inflammatory properties. However, the exact mechanism of action of the devil’s claw phytocomplex remains unclear, and its interaction with mammalian receptors has not been fully investigated. Despite this, studies have shown that it can reduce of COX-2, TNF-a and interleukin-6 [37], suggesting that it may provide anti-inflammatory and analgesic effects [38].

### 4.4. Concerning Non-Cannabinoid Compounds

Pain Relief contains other bioactive compounds beyond cannabinoids, including myrcene, a common monoterpene, found abundantly in plants of the *cannabaceae* family. Unlike other plants, *Cannabis* has undergone unique breeding and selection processes. Due to its recreational use, breeders have aimed to produce strains with high cannabinoid content and appealing flavors, which is why certain terpenes are very prevalent in cannabis plants. Myrcene is also one of the more recently compounds involved in the pain perception mechanism; it participates by regulating the calcium fluxes of TPRV channels, thereby reducing pain perception [39]. In the devil’s claw, the most active constituents are found in the root, primarily iridoid glycosides, which make up approximately 3% of the root’s dry weight [40]. The plant also contains various bioactive molecules, including flavonoids (kaempferol, luteolin), aromatic acids (caffeic acid, chlorogenic acid, cinnamic acid), phytosterols (β-sitosterol, stigomasterol), triterpenes (ursolic and oleanic acid), and harpagoquinone [41]. Indeed, devil’s claw is one of the oldest natural products used to treat pain [16]. Despite the root’s efficacy in treating pain and inflammation-related conditions [41], an effect that likely enhances the anti-inflammatory properties of the tested product, the exact mechanism remains unclear. However, it is thought to involve harpagoside’s role in reducing leukotrienes production [42,43].

## 5. Conclusions

Based on the above results, we can conclude that Pain Relief may reduce pain perception in dogs, thereby enhancing their quality of life, even though the precise biochemical mechanism of its various ingredients is not fully understood. While each ingredient may influence individual inflammatory response and pain perception in dogs, the interactions among these compounds remain unclear.

Further studies are required to better understand the interactions among cannabinoids and between cannabinoids and other natural compounds with similar physiological effects and to learn more about the pharmacokinetics of natural products used in the treatment of chronic pain. Nonetheless, it is evident that these natural compounds hold significant potential as a key resource for future medicine.

## 6. Limitations

The limited number of samples and the relatively short time of administration represent two limitations of this study. Indeed, the effects on pain perception, as well as on oxidative and inflammatory states are clear, suggesting that the supplement might represent an useful tool to act on pain, improving the quality of life of dogs. Studies using a longer treatment with a follow-up of the beneficial effects and its possible usefulness if used in combination with drug therapy remain to be addressed. A potential limitation could arise from product administration by dog owners, leading to an ‘uncontrolled’ dosage. However, dropper dosing is both simple and precise, and the consistency of our results may indicate that the product was administered correctly. Another possible limitation is the inability to determine whether the observed effects are primarily due to a single component or the synergy of various ingredients. Nonetheless, the aim of the study was not to isolate the most effective molecule but to evaluate the overall efficacy of the product.

## Data Availability

The data presented in this study are available on request from the corresponding author.

## References

[1] McMillan F.D. (2019). Mental Health and Well-Being in Animal.

[2] Kitchell R.L., Howard H.E. (2013). Animal Pain: Perception and Alleviation.

[3] Gruen M.E., Lascelles B.D.X., Colleran E., Gottlieb A., Johnson J., Lotsikas P., Marcellin-Little D., Wright B. (2022). AAHA Pain Management Guidelines for Dogs and Cats. J. Am. Anim. Hosp. Assoc..

[4] Mastellone V., Musco N., Vassalotti G., Piantedosi D., Vastolo A., Cutrignelli M.I., Britti D., Cortese L., Lombardi P. (2020). A nutritional supplement (Dìlsh™) improves the inflammatory cytokines response, oxidative stress markers and clinical signs in dogs naturally infected by leishmania infantum. Animals.

[5] Karl T., Garner B., Cheng D. (2017). The therapeutic potential of the phytocannabinoid cannabidiol for Alzheimer’s disease. Behav. Pharmacol..

[6] Stanescu A.M.A., Bejan G.C., Balta M.D., Andronic O., Toma C., Busnatu S. (2024). The Perspective of Cannabidiol in Psoriasis Therapy. Psoriasis.

[7] Smith G.L., Satino J. (2021). Hair Regrowth with Cannabidiol (CBD)-rich Hemp Extract—A Case Series. Cannabis.

[8] Dariš B., Tancer Verboten M., Knez Ž., Ferk P. (2019). Cannabinoids in cancer treatment: Therapeutic potential and legislation. Bosn. J. Basic Med. Sci..

[9] Cahill S.P., Lunn S.E., Diaz P., Page J.E. (2021). Evaluation of Patient Reported Safety and Efficacy of Cannabis From a Survey of Medical Cannabis Patients in Canada. Front. Public Health.

[10] Pero M.E., Meregalli C., Qu X., Shin G.J., Kumar A., Shorey M., Rolls M.M., Tanji K., Brannagan T.H., Alberti P. (2021). Pathogenic Role of Delta 2 Tubulin in Bortezomib-induced Peripheral Neuropathy. Proc. Natl. Acad. Sci. USA.

[11] Pero M.E., Chowdhury F., Bartolini F. (2023). Role of tubulin post-translational modifications in peripheral neuropathy. Exp. Neurol..

[12] Legare C.A., Raup-Konsavage W.M., Vrana K.E. (2022). Therapeutic Potential of Cannabis, Cannabidiol, and Cannabinoid-Based Pharmaceuticals. Pharmacology.

[13] Mechoulam R., Shvo Y. (1963). Hashish—I. The structure of Cannabidiol. Tetrahedron.

[14] McDougall J.J., McKenna M.K. (2022). Anti-Inflammatory and Analgesic Properties of the Cannabis Terpene Myrcene in Rat Adjuvant Monoarthritis. Int. J. Mol. Sci..

[15] Brendler T. (2021). From Bush Medicine to Modern Phytopharmaceutical: A Bibliographic Review of Devil’s Claw (*Harpagophytum* spp.). Pharmaceuticals.

[16] Gxaba N., Manganyi M.C. (2022). The Fight against Infection and Pain: Devil’s Claw (*Harpagophytum procumbens*) a Rich Source of Anti-Inflammatory Activity: 2011–2022. Molecules.

[17] Pollmeier M., Toulemonde C., Fleishman C., Hanson P.D. (2006). Clinical evaluation of firocoxib and carprofen for the treatment of dogs with osteoarthritis. Vet. Rec..

[18] della Rocca G., Schievano C., Di Salvo A., Hielm-Björkman A.K., della Valle M.F. (2024). Psychometric Testing and Validation of the Italian Version of the Helsinki Chronic Pain Index (I-HCPI) in Dogs with Pain Related to Osteoarthritis. Animals.

[19] Millar S.A., Stone N.L., Yates A.S., O’Sullivan S.E. (2018). A Systematic Review on the Pharmacokinetics of Cannabidiol in Humans. Front. Pharmacol..

[20] Musco N., Vassalotti G., Mastellone V., Cortese L., Della Rocca G., Molinari M.L., Calabrò S., Tudisco R., Cutrignelli M.I., Lombardi P. (2019). Effects of a nutritional supplement in dogs affected by osteoarthritis. Vet. Med. Sci..

[21] Pasquini A., Luchetti E., Marchetti V., Luchetti E., Marchetti V., Cardini G., Iorio E.L. (2008). Analytical performances of d-ROMs test and BAP test in canine plasma. Definition of the normal range in healthy Labrador dogs. Vet. Res. Commun..

[22] Belshaw Z., Yeates J. (2018). Assessment of quality of life and chronic pain in dogs. Vet. J..

[23] Phillips D.M. (2000). JCAHO pain management standards are unveiled. Joint Commission on Accreditation of Healthcare Organizations. JAMA.

[24] McGrath S., Bartner L.R., Rao S., Kogan L.R., Hellyer P.W. (2018). A report of adverse effects associated with the administration of cannabidiol in healthy dogs. J. Am. Holist. Vet. Med. Assoc..

[25] Mosley C., Gaynor J., Cital S., Brassard J., Cital S., Kramer K., Hughston L., Gaynor J.S. (2021). Cannabinoids for pain management. Cannabis Therapy in Veterinary Medicine.

[26] Agarwal N., Pacher P., Tegeder I., Amaya F., Constantin C.E., Brenner G.J., Rubino T., Michalski C.W., Marsicano G., Monory K. (2007). Cannabinoids mediate analgesia largely via peripheral type 1 cannabinoid receptors in nociceptors. Nat. Neurosci..

[27] Schaible H.G., Schmidt R.F. (1983). Activation of groups III and IV sensory units in medial articular nerve by local mechanical stimulation of knee joint. J. Neurophysiol..

[28] Davis K.D., Meyer R.A., Campbell J.N. (1993). Chemosensitivity and sensitization of nociceptive afferents that innervate the hairy skin of monkey. J. Neurophysiol..

[29] Hu S., Belcaro G., Cesarone M.R., Feragalli B., Cotellese R., Dugall M., Scipione C., Scipione V., Maione C., Maramaldi G. (2020). A sport cream (Harpago-Boswellia-ginger-escin) for localized neck/shoulder pain. Minerva Med..

[30] Sun D., Li X., Nie S., Liu J., Wang S. (2023). Disorders of cancer metabolism: The therapeutic potential of cannabinoids. Biomed. Pharmacother..

[31] Malheiro R.F., Carmo H., Carvalho F., Silva J. (2022). Cannabinoid-mediated targeting of mitochondria on the modulation of mitochondrial function and dynamics. Pharmacol. Res..

[32] Sermet S., Li J., Bach A., Crawford R.B., Kaminski N.E. (2021). Cannabidiol selectively modulates interleukin (IL)-1β and IL-6 production in toll-like receptor activated human peripheral blood monocytes. Toxicology.

[33] Stone N.L., England T.J., O’Sullivan S.E. (2021). Protective Effects of Cannabidivarin and Cannabigerol on Cells of the Blood-Brain Barrier Under Ischemic Conditions. Cannabis Cannabinoid Res..

[34] Anand U., Oldfield C., Pacchetti B., Anand P., Sodergren M.H. (2021). Dose-Related Inhibition of Capsaicin Responses by Cannabinoids CBG, CBD, THC and their Combination in Cultured Sensory Neurons. J. Pain Res..

[35] Arthur P., Kalvala A.K., Surapaneni S.K., Singh M.S. (2024). Applications of Cannabinoids in Neuropathic Pain: An Updated Review. Crit. Rev. Ther. Drug. Carrier. Syst..

[36] Pagano C., Savarese B., Coppola L., Navarra G., Avilia G., Laezza C., Bifulco M. (2023). Cannabinoids in the Modulation of Oxidative Signaling. Int. J. Mol. Sci..

[37] Fiebich B., Muñoz E., Rose T., Weiss G., McGregor G. (2011). Molecular Targets of the Anti-inflammatory *Harpagophytum procumbens* (Devil’s claw): Inhibition of TNFα and COX-2 Gene Expression by Preventing Activation of AP-1. Phytother. Res..

[38] Rahimi A., Razmkhah K., Mehrnia M., Mohamadnia A., Sahebjamee H., Salehi S., Asl E.A., Tahmasebi H., Shandiz S.A.S., Davouodbeglou F. (2016). Molecular docking and binding study of harpagoside and harpagide as novel anti-inflammatory and anti-analgesic compound from *Harpagophytum procumbens* based on their interactions with COX-2 enzyme. Asian Pac. J. Trop. Dis..

[39] Jansen C., Shimoda L.M.N., Kawakami J.K., Ang L., Bacani A.J., Baker J.D., Badowski C., Speck M., Stokes A.J., Small-Howard A.L. (2019). Myrcene and terpene regulation of TRPV1. Channels.

[40] Baghdikian B., Lanhers M.C., Fleurentin J., Ollivier E., Maillard C., Balansard G., Mortier F. (1997). An analytical study, anti-inflammatory and analgesic effects of Harpagophytum procumbens and Harpagophytum zeyheri. Planta Med..

[41] Burger J.F.W., Brandt E.V., Ferreira D. (1987). Iridoid and phenolicglycosides from Harpagophytum procumbens. Phytochemistry.

[42] Warnock M., McBean D., Suter A., Tan J., Whittaker P. (2007). Effectiveness and safety of Devil’s Claw tablets in patients with general rheumatic disorders. Phytother. Res..

[43] Loew D., Möllerfeld J., Schrödter A., Puttkammer S., Kaszkin M. (2001). Investigations on the pharmacokinetic properties of Harpagophytum extracts and their effects on eicosanoid biosynthesis in vitro and ex vivo. Clin. Pharmacol. Ther..

